# *CLEC16A*—An Emerging Master Regulator of Autoimmunity and Neurodegeneration

**DOI:** 10.3390/ijms24098224

**Published:** 2023-05-04

**Authors:** Rahul Pandey, Marina Bakay, Hakon Hakonarson

**Affiliations:** 1 Center for Applied Genomics, Children’s Hospital of Philadelphia, Abramson Research Center, 3615 Civic Center Boulevard, Philadelphia, PA 19104-4318, USA; pandeyr@chop.edu (R.P.); bakay@chop.edu (M.B.); 2 Department of Pediatrics, The University of Pennsylvania School of Medicine, Philadelphia, PA 19104-4318, USA

**Keywords:** *CLEC16A*, C-type lectin-like domain family 16A (*CLEC16A*) gene, genome-wide association studies (GWAS), autoimmunity, susceptibility loci, autophagy, mitophagy, suppressor of cytokine signaling 1 (SOCS1), neurodegeneration, type 1 diabetes (T1D), Parkinson’s disease (PD)

## Abstract

CLEC16A is emerging as an important genetic risk factor for several autoimmune disorders and for Parkinson disease (PD), opening new avenues for translational research and therapeutic development. While the exact role of CLEC16A in health and disease is still being elucidated, the gene plays a critical role in the regulation of autophagy, mitophagy, endocytosis, intracellular trafficking, immune function, and in biological processes such as insulin secretion and others that are important to cellular homeostasis. As shown in both human and animal modeling studies, CLEC16A hypofunction predisposes to both autoinflammatory phenotype and neurodegeneration. While the two are clearly related, further functional studies are needed to fully understand the mechanisms involved for optimized therapeutic interventions. Based on recent data, mitophagy-inducing drugs may be warranted, and such therapy should be tested in clinical trials as these drugs would tackle the underlying pathogenic mechanism (s) and could treat or prevent symptoms of autoimmunity and neurodegeneration in individuals with *CLEC16A* risk variants. Accordingly, interventions directed at reversing the dysregulated mitophagy and the consequences of loss of function of CLEC16A without activating other detrimental cellular pathways could present an effective therapy. This review presents the emerging role of CLEC16A in health and disease and provides an update on the disease processes that are attributed to variants located in the *CLEC16A* gene, which are responsible for autoimmune disorders and neurodegeneration with emphasis on how this information is being translated into practical and effective applications in the clinic.

## 1. Introduction

*CLEC16A* is implicated in multiple autoimmune diseases and in the pathogenesis of Parkinson’s disease (PD). The shared association of *CLEC16A* in these diverse inflammatory, autoimmune, and neurodegenerative diseases suggests that CLEC16A could be a critical regulator of broad biological processes involving autoimmune responses and neurodegeneration. Mitochondrial dysfunction, persistent ER stress, oxidative stress, inflammation, and altered lipid metabolism, all of which have been attributed to loss of function variants in *CLEC16A*, appear to be the key mechanisms involved in the pathogenesis of these diseases. As a consequence, *CLEC16A* has become an attractive candidate for functional studies to explore the pathogenic mechanisms and potential therapeutic options. Autoimmune diseases develop as a consequence of a synergistic combination of genetic predisposition, largely unknown environmental triggers, and immunologic events. Formerly, *KIAA0350*, now known as *CLEC16A*, was identified as a type 1 diabetes susceptibility locus in 2007 [1]. In addition, GWAS has shown that CLEC16A is associated with multiple autoimmune diseases, including multiple sclerosis, primary adrenal insufficiency, systemic lupus erythematosus, celiac disease, Crohn’s disease, and rheumatoid arthritis, as well as PD. Additional research is required to determine the specific variants that cause the observed effects in the 16p13 gene region. Given the presence of multiple potential effectors and/or modifying genes at the locus, further detailed mapping, and functional investigations of the 16p13 region are warranted.

Studies have shown that CLEC16A is involved in endosomal trafficking [2,3], autophagy [4,5], mitophagy [6], HLA-II antigen presentation [7,8,9], thymic selection [4], and NK cell function [10,11]. A global knockout of CLEC16A in mice leads to abnormal mitophagy, upregulated inflammatory cytokine response, and increased risk of autoimmunity [12]. Interestingly, the knockout mice also develop severe neurological phenotypes with impaired gait and dystonic postures [13]. This is consistent with previous observations in two other mouse strains carrying independent constitutive CLEC16A mutations [14]. Here, we review the emerging role of CLEC16A in autoimmune disorders and neurodegeneration. This article examines the identification of susceptibility variants located in the CLEC16A gene region, as well as the biological function of the CLEC16A. A susceptibility variant is a genetic variation that implies an elevated risk of developing a disease. However, having a genetic susceptibility does not necessarily result in the individual contracting the disease as lifestyle and environmental factors also influence the risk. Additionally, the article discusses recent advancements in the understanding of the implications of these findings to autoimmune disorders and neurodegeneration. Finally, we review opportunities that focus on the development of repurposed drugs with modulatory effects on mitophagy and inflammatory signaling pathways, aiming at compensating for the attenuated CLEC16A activity and presenting future candidate opportunities for targeted interventions involving autoimmune and neurodegenerative disorders, such as PD.

## 2. *CLEC16A* (16p13)—An Autoimmune Candidate Gene

**Figure 1:**
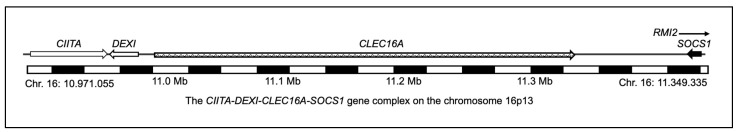
Schematic depicting the chromosome16p13 genetic region comprising *CIITA* (Class II Major Histocompatibility Complex Transactivator), *DEXI* (homolog, cytotoxic suppression), *CLEC16A* (C-type lectin domain family 16, member A), *SOCS1* (Suppressor of Cytokine Signaling 1), and *RMI2* (RecQ Mediated Genome Instability 2). (Genome Reference Consortium Human Build 38 (GRCh38), chromosome 16: 10.971.055–11.349.335).

In 2007, we first identified the region mapping to KIAA0350 (now called C-type lectin-like domain family 16A (*CLEC16A*) as a novel type 1 diabetes (T1D) susceptibility locus within a 238-kb linkage disequilibrium (LD) block located on chromosome 16p13 [1]. The human *CLEC16A* gene consists of 24 exons and encodes three different splice variants, two long isoforms expressed from all 24 exons or from 23 exons, and a shorter 21-exon transcript variant that encodes a protein with a truncated C terminus (https://www.ncbi.nlm.nih.gov/nuccore/NM_015226, accessed on 14 April 2023). The chromosome 16p13.13 locus ~530 kb long region harbors four genes (*CIITA*-*DEXI*-*CLEC16A*-*SOCS1)* (Figure 1). *CLEC16A* is flanked by two neighboring genes: *CIITA*, which is required for the expression of MHC Class II; and *SOCS1*, a negative modulator of cytokine signaling. The 16p13 genetic locus is also comprised of *DEXI*, a gene of unknown function and activated by immune suppressors.

CLEC16A was initially considered to be the primary T1D susceptibility gene within the locus, as most of the disease-associated SNPs reside within its 238 kb gene. There has been debate regarding the causal T1D gene within the chromosome 16p13.13 locus. Functional assessments of DEXI have been limited to date [15,16]. Using the loss of function approach in NOD (non-obese diabetic) mice, Nieves-Bonilla et al. addressed the T1D causality (CLEC16A vs. DEXI) within the 16p13.13 locus [17]. The authors demonstrate that DEXI does not affect the risk of developing diabetes. This suggests that CLEC16A, not DEXI, is the etiological T1D gene within chromosome region 16p13.13. Emerging data further supports the functional and multi-system role of CLEC16A in various cell types of importance to T1D.

The risk loci, *CIITA*-*DEXI*-*CLEC16A*-*SOCS1*, are shared among autoimmune diseases [18,19]. *SOCS1* and *CIITA* have established roles in inflammation and autoimmunity [20,21,22]. SOCS1, a negative modulator of cytokine signaling, is important for immune cell homeostasis and the regulation of inflammation [23,24]. Gene variants in the 5’ untranslated region (UTR) of *SOCS1* (rs243324 and rs441349) have been identified in cytokine pathway gene screenings as multiple sclerosis (MS) susceptibility variants [19,25]. Many questions still remain unanswered regarding the specific molecular mechanisms and tissue-specific contributions by which CLEC16A contributes to autoimmunity. The emerging role of CLEC16A in autophagy, mitophagy, and immune regulation makes it an ideal candidate to be explored as a potential druggable target in *CLEC16A*-associated pathologies.

CLEC16A belongs to the C-type lectin (CLEC) protein family. CLEC proteins play a crucial role in regulating adaptive immune responses by identifying antigens through their carbohydrate recognition domain and transporting them to the surface of antigen-presenting cells (APCs) via the endosomal system. However, unlike other CLEC proteins, CLEC16A does not have an active or full-length carbohydrate recognition domain. Instead, it functions as an E3-ubiquitin ligase and is involved in regulating autophagy [3] and mitophagy [6]. The full-length *CLEC16A* gene encodes a large 1053 amino acids protein. Besides the atypical C-type lectin-like domain (CTLD), CLEC16A protein contains an immunoreceptor tyrosine-based activation motif (ITAM), a trans-membrane (TM) region, and an *N*-terminal highly conserved and uncharacterized FPL motif [26]). Recently, it was discovered that the C terminus of CLEC16A, which does not share homology with known protein domains, is an intrinsically disordered protein region (IDPR) [27]. IDPRs are known to support critical biological functions, including signal transduction, protein complex assembly, and protein stability [28,29]. However, their mechanistic roles in disease are still poorly understood. Recent studies reported that the CLEC16A’s C-terminal IDPR is critical for the assembly of the mitophagy regulatory machinery, and in-vivo loss impairs mitochondrial function and glucose-stimulated insulin secretion [27]. This implies that the pathogenic human gene variants that disrupt IDPRs are novel contributors to diabetes and CLEC16A-associated diseases.

## 3. CLEC16A in Health and Diseases through GWAS

The landscape of genetic research for complex common diseases underwent a drastic transformation in the mid-2000s with the introduction of genome-wide association studies (GWAS), which significantly accelerated the pace and efficiency of identifying susceptibility loci. The breakthrough was laid by the HapMap project [30,31]. By testing up to millions of variants in a hypothesis-free context, GWAS has empowered scientists to discover novel susceptibility loci for human diseases, including cancer, neurodegenerative, cardiac, infectious, inflammatory, and autoimmune diseases.

The first full-scale GWAS for T1D was published by our group [1] and the Wellcome Trust Case-Control Consortium (WTCCC) simultaneously [32]. We identified *CLEC16A* as a novel T1D susceptibility gene and reported three common non-coding variants that reached genome-wide significance (rs2903692, rs725613, and rs17673553). This finding was confirmed for T1D in the European descent populations [33,34]. To date, several SNPs within the *CLEC16A* gene show association with T1D in several populations such as Sardinian [35], Spanish [36], south-east USA [37], Chinese [38,39], Japanese [40], and adult-onset of autoimmune diabetes [41].

**Figure 2:**
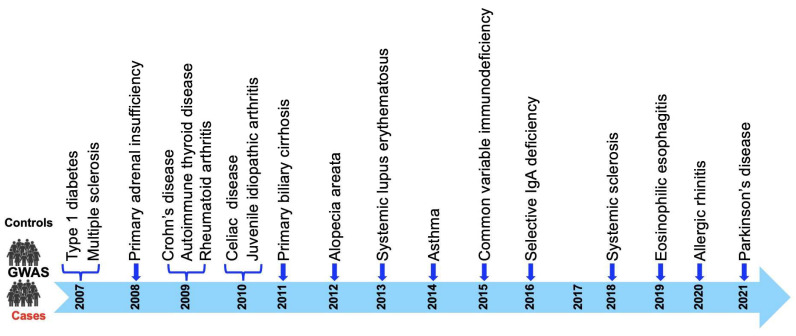
*CLEC16A* disease association timeline. *CLEC16A* association with 18 diseases described to date: T1D was the first disease to show genome-wide association (GWA) with *CLEC16A*, in 2007, followed by MS. Parkinson’s disease is the latest disease to be associated with *CLEC16A*, reported in 2021.

The *CLEC16A* locus is now associated with the susceptibility to 18 autoimmune diseases (Figure 2) depicted in a timeline through GWAS and meta-analysis, including type1 diabetes (T1D) [1,32,33,34,35,36,38,39,40,41,42,43,44,45], multiple sclerosis (MS) [18,19,35,36,46,47,48,49,50,51,52,53,54,55,56,57,58], primary adrenal insufficiency (PAI) [59,60], systemic lupus erythematosus (SLE) [61,62,63,64], Crohn’s disease (CD) [65], selective immunoglobulin A deficiency (IgA) [66], alopecia areata (AA) [7,67], juvenile idiopathic arthritis (JIA) [68], rheumatoid arthritis (RA) [36,68], primary biliary cirrhosis (PBC) [69,70,71,72], asthma [73,74,75,76,77,78,79], allergic rhinitis (AR) [80,81], autoimmune thyroid diseases (ATD) [42,82], common variable immunodeficiency (CVID) [83], eosinophilic esophagitis (EE) [84], juvenile idiopathic arthritis (JIA) [68], selective IgA deficiency [66], Celiac disease [85], systemic sclerosis [86], and Parkinson’s disease (PD) [87,88].

Recent GWAS has associated *CLEC16A* with Parkinson’s disease, a well-characterized neurodegenerative disorder [87,88]. Parkinson’s is the second most common neurodegenerative disorder characterized by the loss of dopaminergic neurons in substantia nigra pars compacta causing clinical symptoms such as tremor, rigidity, bradykinesia, and postural instability. The majority of PD cases are sporadic and only about 15% of people with Parkinson’s have a family history of this disorder. Several monogenic forms of PD have been reported, including numerous genetic risk factors increasing the risk to develop PD. Familial cases of PD are caused by mutations in the *SNCA* (PARK1) [89], *Parkin* (PARK2) [90], *DJ-1* (PARK7) [91], *PINK1* (PARK6) [92], *LRRK2* (PARK8) [93,94], *ATP13A2* (PARK9), *VPS13C* [95,96,97] and *GBA* [98], all well-established single gene disorders. Most people develop the disease after age 60; however, 5–10% experience onset before the age of 50. Early-onset Parkinson’s disease (EOPD) is a mitochondrial disease (Nigral Mitochondrial Disease) caused by a loss of function mutations in the genes encoding PINK1 and Parkin. Both enzymes are functionally linked and together direct a neuroprotective mitochondrial quality control ensuring the elimination of damaged organelles from cells via the autophagy-lysosome system (i.e., mitophagy), which is lost in early-onset PD. Loss of function variants in *CLEC16A* show comparable neurodegenerative effects in our modeling studies as a consequence of mitochondrial death due to mitophagy/autophagy dysfunction [13]. The mitochondrial dysregulation results from loss of Parkin regulation, where CLEC16A interaction with NRDP1 is disrupted due to mutation variants in CLEC16A, and as a result, NRDP1’s regulation of keeping Parkin in check is lost with resulting mitochondrial death and subsequent mitophagy/autophagy dysfunction.

The identification of PD risk factors using classical methods such as gene mapping or candidate gene approaches is time-consuming and difficult. GWAS presents a hypothesis-free technology to facilitate gene discovery. A recent GWA study highlights 11 SNPs associated with PD mapped to genes (*MS4A4E*, *DKKL1*, *MPV17L2*, *MIR499A*, *AGAP2*, *CLECL1*, *CLEC16A*, *MIR196A2*, *IL7R*, *INPP5D*, and *ZSWIM4*) [87]. These genes encode proteins essentially involved in pathways related to PD, including neuroinflammation, peripheral immune response, apoptosis, endo-lysosomal system, mitochondrial function/morphology in humans and animal models, axon guidance, and autophagy. Fan et al. reported CLEC16A rs6498169 and rs7200786 to be associated with PD in the Han Chinese population [88]. The quantitative trait locus analysis suggests that the expression of CLEC16A could be influenced in two different ways. Specifically, rs6498169 may affect the expression by regulating splicing, while rs7200786 could affect both the expression and splicing of CLEC16A. GWAS PD association is supported by evidence of Parkin mutations in early-onset PD, the presence of α-synuclein-reactive T cells, and an association between their reactivity and preclinical/early PD, pointing to a connection between PD and autoimmunity [99].

**Figure 3:**
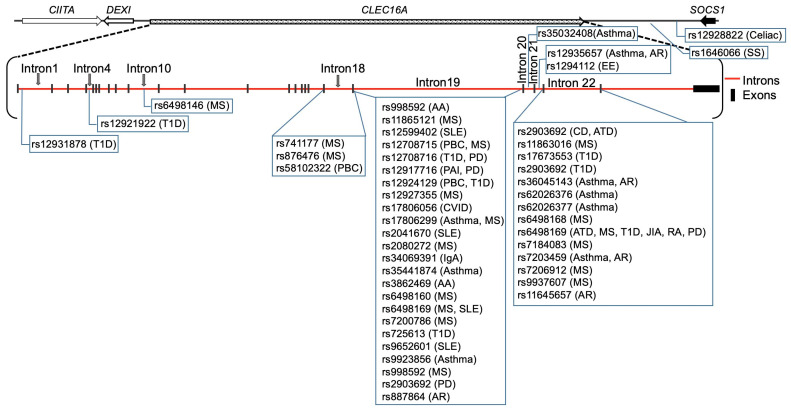
The 18 autoimmune disease-associated single nucleotide polymorphisms (SNPs) and their localization are depicted on the 238 kb *CLEC16A* gene. The chromosome 16p13.13 locus ~530 kb long region is the house of four genes (*CIITA*-*DEXI*-*CLEC16A*-*SOCS1*). *CLEC16A* is flanked by two neighboring genes: *CIITA*, which is required for the expression of MHC Class II, and *SOCS1*, a negative modulator of cytokine signaling. Primary adrenal insufficiency (PAI), allergic rhinitis (AR), alopecia areata (AA), asthma, autoimmune thyroid diseases (ATD), Celiac disease, common variable immunodeficiency (CVID), Crohn’s disease (CD), eosinophilic esophagitis (EE), juvenile idiopathic arthritis (JIA), multiple sclerosis (MS), Parkinson’s disease (PD), primary biliary cirrhosis (PBC), rheumatoid arthritis (RA), selective IgA deficiency, systemic lupus erythematosus (SLE), systemic sclerosis, and type 1 diabetes (T1D).

In-vivo studies have demonstrated that PINK1/Parkin-regulated mitophagy can curtail innate immune responses, but if mitophagy malfunctions, it may enhance the STING pathway, resulting in an inflammatory phenotype that could eventually contribute to the loss of dopaminergic neurons [100]. It is worth highlighting the patients with Parkin mutations also exhibit components of innate immunity activation [100]. Parkin is a ubiquitin E3 ligase. Recent research suggests that mitochondrial dysfunction and immune responses are linked [13,101], implying the possibility of shared genetic pathways in the pathogenesis of Parkinson’s disease (PD) and autoimmune diseases (AIDs) [99,102]. Mitophagy impairment results in the progressive accumulation of defective mitochondria, leading to neuronal death and eventual neurodegeneration. CLEC16A is known to play a critical role in Nrdp1-PINK-Parkin-mediated mitophagy [6] and autophagy [2,3], and alterations in the CLEC16A expression may result in dysregulated/deficient mitophagy, increasing the likelihood of Parkinson’s disease. *CLEC16A* association to date with 18 autoimmune diseases, supports the hypothesis of shared pathways of autoimmune susceptibility. Figure 3 and Table 1 summarize the *CLEC16A* SNPs associated with all disorders reported to date in alphabetical order.

## 4. Biological Role of CLEC16A

### 4.1. Autophagy Regulation

CLEC16A has been found to play a role in the regulation of autophagy, an evolutionarily conserved process by which cells break down and recycle damaged or dysfunctional cellular components [3]. Autophagy is important for maintaining cellular homeostasis, and dysregulation of this process has been implicated in various diseases, including neurodegeneration, cancer, metabolic disorders, and autoimmunity [106,107,108]. Based on degraded cell components, some selective types of autophagy identified are mitophagy, ribophagy, reticulophagy, lysophagy, pexophagy, lipophagy, and glycophagy [106]. The regulation of autophagy is intricate and depends on several factors such as nutrient availability, cellular stress, energy status, a complex network of signaling pathways, and cellular factors. One of the primary negative regulators of autophagy is the mammalian target of the rapamycin (mTOR) signaling pathway. The activation of mTORC1 is triggered by various stimuli, including those mediated by growth factors, nutrients, and amino acids, as well as tuberous sclerosis complex 1/2-dependent and independent mechanisms [109,110,111]. The activity of mTOR is closely linked to fundamental cellular processes, including cell proliferation, differentiation, apoptosis, and autophagy. The understanding of the molecular mechanism that regulates the different phases of the autophagic process and its role in the development of diseases is still being explored.

The first evidence of CLEC16A biological function came from *Drosophila* (*Drosophila melanogaster*) ortholog-*Ema*, an endosomal protein known to promote autophagosomal growth and function through interactions with the class C Vps-HOPS complex, suggesting that the regulation of autophagosome morphogenesis may be a fundamental function of this gene family [2]. The *ema* mutant showed enlarged endosomal compartments with failed endosomal maturation and inhibition of lysosomal degradation. The human orthologue of *ema*, CLEC16A, rescued the Drosophila mutant demonstrating conserved function [2,3] for this protein.

The role of CLEC16A in immune cells was reported initially for T cells in MS [21]. The study explored the role of the different MS-associated SNPs (rs12708716, rs6498169, and rs7206912) within the 16p13 chromosomal region harboring the *CIITA*-*DEXI*-*CLEC16A*-*SOCS1* gene complex in the whole-blood and thymic samples and suggested a possible regulatory role for non-coding *CLEC16A* SNPs and a common control mechanism for the expression of CLEC16A, SOCS1, and DEXI. Later, the same group reported *CLEC16A* polymorphism in intron 19 to be associated with higher expression of CLEC16A in CD4^+^ T cells [112]. CLEC16A was subsequently reported to be specifically located in Rab4a-positive recycling endosomes in T cells [113]. Knocking down CLEC16A in Jurkat cells led to reduced expression of the T cell receptor on the cell surface. However, this did not significantly affect the T cell activation response in-vitro in Jurkat cells or primary CD4(+) T cells in humans [113]. The role of CLEC16A through these studies in T cells is still enigmatic. 

Another study coupled MS-associated genes to the regulation of human leukocyte antigen class II (HLA-II) with strong upregulation of CLEC16A. Mechanistically, CLEC16A participated in the molecular machinery of HLA-II positive late endosome formation and trafficking to perinuclear regions, involving the dynein motor complex and interaction with the HOPS complex, highlighting its role in recycling and late endosomal trafficking [9]. Endosomes are known to recycle the cargo to the plasma membrane via the trans-Golgi network or direct to late endosomes for degradation. CLEC16A serves as a direct regulator of the human leukocyte antigen class II pathway in antigen-presenting cells. 

Schuster et al. utilized a lentiviral knock-down (KD) technique in the non-obese diabetic (NOD) mice model to demonstrate that inhibiting CLEC16A via silencing can protect against autoimmunity in T1D, which stands in contrast to the increased susceptibility to autoimmunity caused by reducing the expression of CLEC16A. In this study, disease protection was attributable to T cell hyporeactivity, which was secondary to changes in thymic epithelial cell (TEC) stimuli that drive thymocyte selection. The study indicated that T cell selection and reactivity were impacted by CLEC16A variation in thymic epithelium owing to CLEC16A’s role in TEC autophagy [4].

**Figure 4:**
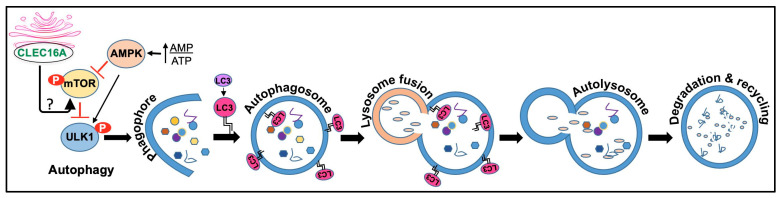
CLEC16A functions in autophagy. Autophagy comprises initiation, membrane nucleation, phagophore formation, phagophore expansion, fusion with the lysosome, degradation, and recycling. Initiation: Autophagy is initiated by the formation of a phagophore, a double-membraned structure that forms around the targeted cellular component. Once the phagophore is formed, it elongates and expands, forming the autophagosome. This process is mediated by the ATG12-ATG5-ATG16L complex and the LC3 protein, which is then lipidated and inserted into the growing autophagosome membrane. Maturation: The autophagosome then fuses with a lysosome, forming an autolysosome. The lysosome contains digestive enzymes that break down the contents of the autophagosome, allowing for the recycling of cellular components. Degradation: The degraded components are then released back into the cytoplasm for reuse by the cell. AMPK, mTOR, and ULK complex are all involved in the regulation of autophagy. AMPK (AMP-activated protein kinase) is a key energy-sensing enzyme that is activated in response to energy depletion. Activated AMPK stimulates autophagy by phosphorylating and activating ULK1, a protein involved in the initiation of autophagy. Autophagy induction is known to be inhibited by CLEC16A. Under starvation, CLEC16A clusters in the Golgi to interact with an unidentified mediator that would activate the mTORC1 complex fully once nutrients are available. It is speculated that CLEC16A regulates mTOR by controlling the stability and/or degradation of Rheb, which requires further investigation.

More recently, several studies described the role of CLEC16A in autophagic processes. CLEC16A was shown to have an inhibitory role in starvation-induced autophagy in human cells using ectopic expression and siRNA silencing, via delaying mTOR activation [5]. Cells that overexpressed CLEC16A became more sensitive to nutrient availability, leading to increased mTOR activity, which subsequently reduced LC3-mediated autophagy during nutrient deprivation. On the other hand, the absence of CLEC16A resulted in delayed mTOR activity upon nutrient sensing, leading to enhanced autophagy. CLEC16A was found in cytosolic vesicles and the Golgi, and the removal of nutrients resulted in stronger clustering within the Golgi, possibly positioning it advantageously to activate mTOR upon nutrient replenishment [5]. These findings suggest that CLEC16A located in the Golgi negatively regulates autophagy by modulating mTOR activity as depicted in Figure 4.

Another MS study investigated the role of CLEC16A in B cells. CLEC16A was co-expressed with surface class II-associated invariant chain peptides (CLIP) in human EBV-positive B cell lines (Raji B cells). Stable knockdown of *CLEC16A* in EBV-positive Raji B cells resulted in an upregulation of surface HLA-DR and CD74 (invariant chain), whereas CLIP was slightly but significantly reduced. However, in primary B cells, *CLEC16A* was only induced under CLIP-stimulating conditions in vitro and was predominantly expressed in CLIP^high^ naive populations suggesting that CLEC16A participates in the B Cell Receptor (BCR)-dependent HLA-II pathway in human B cells and that this regulation is impaired during MS disease onset [8].

GWAS have demonstrated *CLEC16A* association with alopecia [7,67]. A recent study showed the importance of autophagy in alopecia areata (AA). Alopecia is considered an organ-specific autoimmune disease of the hair follicle with genetic background and involves an aberrant immune attack on the hair follicle resulting in hair loss. Impairment in autophagy has been implicated in the loss of immune tolerance in human AA [114]. Alopecia GWAS identified *CLEC16A*, STX17, and *BCL2L11/BIM* as risk factors. Using a C3H/HeJ mouse model, the authors showed the accumulation of the autophagy protein SQSTM1 in the hair follicles of AA mice. The C3H/HeJ mouse is an inbred laboratory strain that spontaneously develops an adult-onset disease that resembles adult-onset alopecia areata in humans [115]. p62/SQSTM1 was not altered in immune cells, suggesting that autophagic activity is inhibited in the hair follicles of AA mice. The protein Sequestosome 1 (p62/SQSTM1) is a classical selective autophagy receptor [116]. The inhibition of autophagy results in the accumulation of p62. The induction of autophagy with Tat-BECN1 peptide attenuated alopecia, while treatment with the autophagy blocker chloroquine promoted the disease, compared to untreated mice. Together, these findings suggest the involvement of impaired autophagy in the disease pathogenesis of alopecia [117]. By modulating autophagy, it may be possible to prevent or treat autoimmune and neurodegenerative disorders associated with CLEC16A dysfunction. However, the delineation of the molecular underpinnings and the identification of genetic and/or pharmacological interventions are still needed to develop safe and effective therapies.

### 4.2. Regulator of Mitophagy

Mitophagy, an evolutionary conserved cellular self-degradation of damaged or dysfunctional mitochondria, is crucial for mitochondria quality control and renewal, providing energy to cells, promoting neuronal survival, and supporting overall health. In contrast, impaired mitophagy can lead to the buildup of damaged mitochondria and disrupt cellular function, thereby contributing to the aging process and increasing the risk of neurodegenerative diseases associated with aging [118]. Thus, a better understanding of mitochondrial turnover mechanisms is a key requirement for the development of more efficient therapeutic strategies to battle numerous pathological conditions in humans. Cells possess several mitophagy mechanisms, and different stresses promote mitophagy in distinct cellular contexts. Mitophagy can be categorized into Parkin-dependent and Parkin-independent pathways, which may interact with each other to some extent [119,120,121].

We demonstrated the connection between CLEC16A and pancreatic β-cell function through mitophagy [6]. CLEC16A is a membrane-associated endosomal protein that interacts with E3 ubiquitin ligase Nrdp1. A pancreas-specific deletion of *CLEC16A* led to an increase in Parkin, the master regulator of mitophagy. Islets of mice with this pancreas-specific deletion *(CLEC16A^Δpanc^*) depict abnormal mitochondria with reduced oxygen consumption. Patients with the *CLEC16A* T1D risk allele, rs12708716 G, have reduced expression of CLEC16A in islets and attenuated insulin secretion, suggesting that CLEC16A controls β-cell function and prevents diabetes by controlling mitophagy. We postulate that a defect in insulin secretion is secondary to disrupted autophagy and would predispose beta cells to the autoimmune destruction that causes type 1 diabetes.

**Figure 5:**
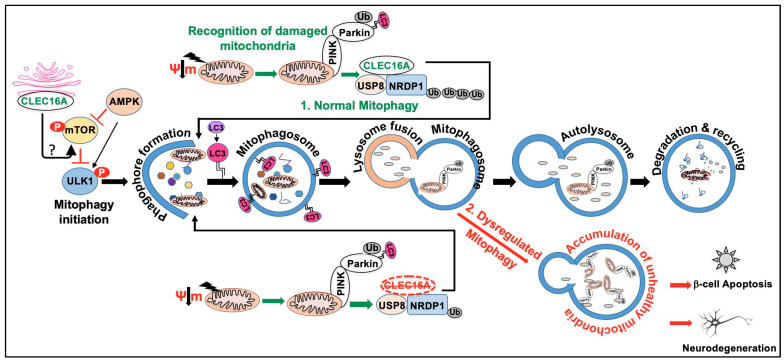
CLEC16A function in mitophagy. Mitophagy is a form of selective autophagy that targets damaged mitochondria for degradation via either the Parkin-dependent or Parkin-independent pathways. Autophagy receptors (P62, NBR1, NDP52, OPT), Ubiquitin, and ubiquitin-binding proteins are involved in the Parkin/PINK1-dependent mitophagy. Parkin-independent mitophagy involves the use of multiple mitophagy receptors (BNIP3, Nix/BNIP3L, FUNDC1, BCL2L13, and FKBP8) to target damaged mitochondria to the LC3-mediated autophagy machinery for clearance. The schematic depicts Parkin/PINK1-dependent mitophagy. AMPK, mTOR, and ULK complexes are also involved in the regulation of mitophagy. The normal mitophagy process comprises mitophagy initiation, membrane nucleation, phagophore formation, recognition of damaged mitochondria, phagophore expansion to form mature mitophagosome, fusion with the lysosome, and degradation (1). CLEC16A hypofunction leads to dysregulated autophagy and the accumulation of unhealthy mitochondria (2). Maintenance of the CLEC16A-NRDP1-USP8 mitophagy complex is crucial for promoting optimal cellular respiration and insulin secretion. Dysregulated mitophagy can lead to a buildup of damaged mitochondria and an increase in oxidative stress, ultimately leading to β-cell dysfunction and death. Similarly, in neurodegenerative diseases such as Parkinson’s, dysregulated mitophagy can lead to an accumulation of damaged mitochondria and impaired energy metabolism in neurons. This can contribute to neuronal dysfunction and death, as well as the accumulation of toxic protein aggregates that are characteristic of these diseases.

CLEC16A is an E3 ubiquitin ligase, along with RNF41/NRDP1 and USP8, which forms a tripartite complex and relies on ubiquitin for its function [122]. Maintenance of the CLEC16A-NRDP1-USP8 mitophagy complex is crucial for promoting optimal cellular respiration and insulin secretion. However, when faced with metabolic stressors associated with diabetes, such as high levels of glucose and fatty acids, the CLEC16A-RNF41-USP8 complex becomes destabilized and can lead to the death of β-cells. Another recent study additionally confirmed the above interaction of CLEC16A with NRDP1 also known as RNF41 [123]. CLEC16A-mediated mitophagy modulation is depicted in Figure 5.

Work from our ubiquitous whole-body, inducible CLEC16A knockout mice (*CLEC16A^ΔUBC^*) revealed that incomplete mitophagy predisposes mice to a cascade of altered immune functions leading to pathogenic inflammation with hyperactive NK cells under dysregulated mitophagy settings [10]. We also showed that CLEC16A restrains natural killer (NK) cell function using an overexpression YTS NK cell line and *CLEC16A^ΔUBC^* mice, indicating that a delicate balance of *CLEC16A* is needed for NK cell function and homeostasis, including cytokine release and cytotoxicity [11].

The NK cell lines, NKL and YTS, were used to assess the biological function of CLEC16A since they are homozygous for the protective (A/A) and non-protective (G/G) alleles (rs2903692-A), respectively. As expected, NKL had high CLEC16A expression and low cytotoxicity compared to YTS. Since *CLEC16A* SNPs were linked to protection against type 1 diabetes and NK cells have been associated with multiple autoimmune diseases, investigating the role of CLEC16A in NK cells could provide new insights into disease pathogenesis. While previous studies suggested that CLEC16A was a membrane-associated protein [2,3,6], our results indicate that it is a cytosolic protein in NK cells, which is consistent with its function to restrain cytolytic activity and cytokine secretion [11]. Protein expression analysis of CLEC16A in human immune cell types shows that B cells have the highest expression, followed by NK and T cells, results that are in concordance with the available microarray data [124,125]. This suggests that CLEC16A expression and action may be relevant in these cell types. NK cells can contribute both directly and indirectly to tissue and organ damage in autoimmune disorders due to their cytotoxic and cytokine-producing abilities. The essential role of bi-directional crosstalk between NK cells and dendritic cells (DCs) during immune responses has been elucidated in recent years [126,127,128], with further investigation ongoing. Disrupted mitophagy settings can lead to altered immune cell populations, increased killing, and upregulated cytokine/chemokine secretion, with a resulting imbalance in DC subsets, which may result in excessive cell death and tissue destruction. This may reflect the inflammatory mechanism involved in the development, progression, and pathogenesis of various autoimmune and inflammatory diseases. Dead cells constitute a source of novel antigens that can further provoke the autoimmune response. As a consequence, the resulting activation and polarization of innate and adaptive immune cells towards a Th-1-type immune response may be among the key factors contributing to the pathogenesis. The fact that conditional targeting of T cells in CD4 Cre *CLEC16A*^ΔUBC^ mice revealed no variation in T cell repertoire and pathological phenotype [11], further reinforces this concept. Previous research has investigated the role of CLEC16A in T-cell co-stimulation and activation in human lymphoblastoid cell lines (LCLs) in dendritic and B cells, which are professional antigen-presenting cells (APCs) and CLEC16A high expressers. The study reported that *CLEC16A* knockdown does not affect T cell activation or proliferation following co-culture with KD or control LCLs [129], suggesting that additional studies on CLEC16A, accounting for its ER localization, are needed to uncover its biological role in other immune cell types. The derailed functional link between CLEC16A and NK cells may further explain the immune dysregulation under disrupted mitophagy conditions in immune cells [10] and the risk of autoimmunity associated with specific *CLEC16A* variants. 

**Figure 6:**
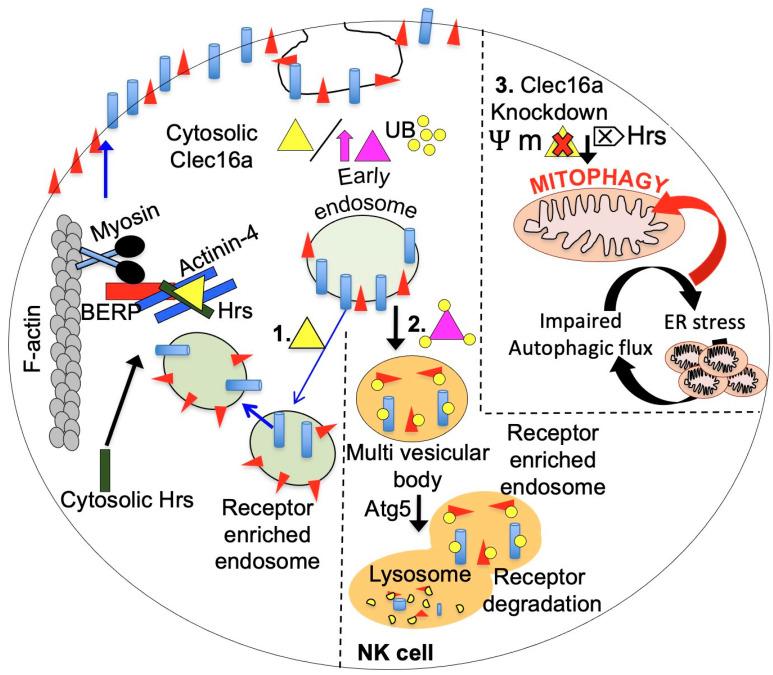
The multifaceted role of CLEC16A in receptor recycling in NK cells is illustrated in a schematic diagram, emphasizing the delicate balance required for NK cell function and homeostasis. The amount of CLEC16A protein is critical for maintaining this balance, as illustrated. In the left panel (1), the normal level of endogenous CLEC16A (yellow triangle) is shown to regulate receptor trafficking by participating in endosome recycling. CLEC16A binds to the Hrs/Actinin-4/BERP/Myosin V (CART) complex, facilitating the transport of receptor-enriched endosomes to the cell surface membrane. The amount of CLEC16A protein serves as a checkpoint for NK cells. Overexpression of CLEC16A (purple triangle, middle panel, 2) leads to its own ubiquitination, resulting in increased association with the CART complex, thereby disrupting efficient receptor recycling. Consequently, receptors are targeted for degradation through autophagy, leading to attenuated receptor signaling. Loss of CLEC16A (right panel, 3) leads to disrupted mitophagy and the accumulation of unhealthy mitochondria. CLEC16A stabilizes and prevents the proteasomal degradation of Nrdp1, which limits the recruitment of Parkin to the mitochondrial surface and promotes autophagosome-lysosome fusion during the late stages of mitophagy.

While various models in different cell types can be proposed for the function of CLEC16A, our findings from the overexpression system in NK cells and knockout mouse model studies indicate that CLEC16A has inherent E3 ligase activity and can undergo self-ubiquitination to regulate receptor turnover and expression through the C Vps-HOPS complex, CART, and autophagy [11] (Figure 6).

Recently, we reported an autoimmune/inflammatory and lipodystrophic phenotype using a whole-body, inducible *CLEC16A*^ΔUBC^ mouse model [12]. Loss of CLEC16A function leads to a vicious cycle of autophagic impairment, ER stress, and activation of lipolytic cascade resulting in loss of all forms of white adipose tissue under dysregulated mitophagy settings. KO mice undergo rapid weight loss, exhibit abnormal lipid metabolism, significantly reduced circulating insulin levels in the presence of normal food consumption, elevated antibody levels, robust cytokine-mediated inflammatory response, and lipodystrophic phenotype. Unlike other established models of lipodystrophy, our *CLEC16A*^ΔUBC^ mice do not exhibit hypertriglyceridemia and hepatic steatosis, implying a novel phenotype different from known mammalian models of lipodystrophy. Treatment of *CLEC16A*^ΔUBC^ mice with the pan-JAK/STAT inhibitor tofacitinib partially rescues the pathological lipodystrophic phenotype and improves survival [12]. Tofacitnib exerts its multifaceted effect on AMPK, mTOR, HSL, autophagy, and JAK-STAT-mediated SOCS signaling and partially reduces the lipodystrophic phenotype. These findings further strengthen the link between *CLEC16A*, mitochondria, metabolism, and immune system perturbations and suggest SOCS signaling to be explored as a potential druggable target in *CLEC16A*-associated pathologies.

## 5. CLEC16A in Neurodegeneration

The genetic linkage of the *CLEC16A* locus to multiple sclerosis was established in 2007 [46], whereas the role of the gene in the nervous system remained unidentified. The initial research on CLEC16A suggested its potential significance in host defense through astrocyte-mediated immune response [130]. The expression of CLEC16A was significantly increased in active astrocytes located in the inflamed cerebral cortex and suggested that the upregulation of CLEC16A could play a role in the activation. Knock-down of CLEC16A in cultured primary astrocytes by siRNA showed that CLEC16A was required for the activation of astrocytes induced by LPS [130]. In 2016, the study utilized two mouse strains carrying independent *CLEC16A* mutations and reported a neurodegenerative disease characterized by motor impairments and loss of Purkinje cells [14]. Homozygous mice carrying a gene-trap insertion in *CLEC16A* (*CLEC16A*^GT/GT^) develops an overt neurological phenotype at 7–8 weeks of age, ascribed to the loss of cerebellar Purkinje cells. *CLEC16A*^curt/curt^ mice, with a 4-nucleotide deletion allele in exon 21, resulting in a frameshift mutation, also lose cerebellar Purkinje cells. The neurons derived from *CLEC16A*-mutant mice demonstrated an increased expression of p62, an autophagy substrate, as well as an accumulation of abnormal intra-axonal membranous structures that bore the autophagy protein LC3, and an unusual Golgi morphology. Despite normal functionality of multiple aspects of endocytosis, lysosome, and Golgi function, the CLEC16A-deficient murine embryonic fibroblasts and HeLa cells showed abnormal bulk autophagy, despite the normal autophagosome formation. The cultured CLEC16A-deficient cells revealed a striking accumulation of LC3 and LAMP-1 positive autolysosomes that contained undigested cytoplasmic contents. Purkinje cell survival necessitates CLEC16A as vital for autolysosome function and clearance.

Recently, we showed that whole-body knockout of *CLEC16A* (*CLEC16A*^ΔUBC^) in adult mice results in an overt neurological phenotype associated with loss of primary sensory axons and loss of Purkinje cells [13]. These results are similar to the observations in *CLEC16A*^GT/GT^ and *CLEC16A*^curt/curt^ mice [14]. It remains to be determined whether primary sensory neurons are affected in these CLEC16A mouse models. Our experiments with *CLEC16A*^ΔUBC^ homozygous mice revealed that they have a severe phenotype and do not survive beyond embryonic stages [13]. In comparison, *CLEC16A*^GT/GT^ mice have a mild and delayed behavioral phenotype and longer survival, while *CLEC16A*^curt/curt^ mice exhibit prenatal/perinatal death, skeletal abnormalities, and decreased body weight. Based on these observations, we can conclude that the *CLEC16A*^GT^ allele likely results in partial loss of function. Our study also found astrogliosis and activated microglia in regions with axonopathy, and indications of dysregulated autophagy in neural regions with and without axonopathy. Moreover, we observed changes in proteins related to mitochondrial oxidative phosphorylation, endoplasmic reticulum stress markers, and ISG15 in neuronal tissues [13]. ISG15 is a ubiquitin-like modifier that inhibits polyubiquitination and is involved in a process called ISGylation, which controls protein aggregate clearance in autophagy [131]. ISG15 activation occurs after cell stress [132] and neuronal damage [133], and is thought to be a potential cause of defective mitophagy in neurodegenerative diseases [134]. ISG15 and ISGylation also control mitochondrial oxidative phosphorylation and recycling in bone marrow-derived macrophages [135]. ISG15 conjugation to Parkin enhances its E3 ubiquitin ligase activity, an example of how ISGylation impacts mitochondrial processes in neurodegeneration [136]. Free ISG15 stimulates IFN-γ production [137]. We speculate that ISG15 is likely to be a key player in linking CLEC16A to downstream autoimmune inflammatory processes due to its numerous roles in ubiquitination and inflammatory processes. Thus, our *CLEC16A*^ΔUBC^ homozygous mice provide a valuable murine model to elucidate these issues in sensory ataxia as well as mitochondrial function in neurodegeneration. 

A recent study reported the first bi-allelic loss-of-function (LoF) *CLEC16A* truncating variants in siblings from two unrelated families with a severe neurodevelopmental disorder, including microcephaly, brain atrophy, corpus callosum dysgenesis, growth retardation, and hypotonia with severe developmental delay [123]. Using zebrafish as an in vivo model, the study shows that the loss of CLEC16A disrupts both autophagy and mitophagy, already during brain development. Additionally, using an in vitro HEK293T cell system, the study demonstrates that CLEC16A closely interacts with retromer components and regulates endosomal fate by fine-tuning levels of TRIM27 and polymerized F-actin on the endosome surface. Dysregulation of CLEC16A-mediated endosomal sorting is associated with neurodegeneration, but it also causes the accumulation of autophagosomes and unhealthy mitochondria during brain development. These findings are in concordance with the observed neurological phenotype in our *CLEC16A*^ΔUBC^ homozygous mice and suggest a pleiotropic role of CLEC16A in the regulation of autophagy, mitophagy, and further support the emerging role of CLEC16A in neurodegeneration.

## 6. Therapeutic Targeting of CLEC16A

**Figure 7:**
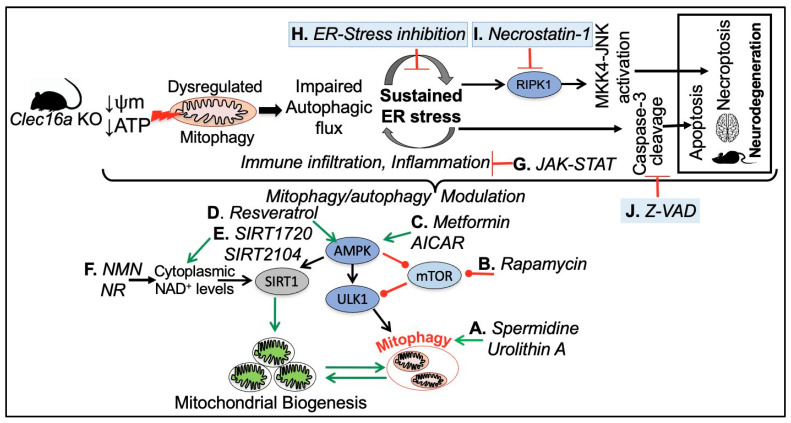
A hypothetical model for *CLEC16A* hypofunction and rescue by mitophagy/autophagy modulation. CLEC16A hypofunction leads to a vicious cycle of mitophagy/autophagy impairment and sustained ER stress. To restore cellular homeostasis in response to mitochondrial dysfunction mTOR, AMPK, and SIRT1 UPR-ER stress pathways, SOCS-mediated JAK-STAT signaling, apoptosis, and necro-apoptosis pathways can be targeted.

CLEC16A is emerging as an important genetic risk factor for several autoimmune diseases and Parkinson’s disease and has opened new avenues for research. While there are no specific therapeutic agents currently that target CLEC16A, there are several drugs that have the potential to benefit patients with CLEC16A dysfunction. Given the complexity and overlapping pathways, combinatorial drug therapy may be required. The above-mentioned studies highlight the involvement and dysregulation of autophagy, mitophagy, and dysregulated SOCS1 signaling. Emerging evidence from our whole-body, inducible mouse models suggests that Clec16A hypofunction, at least in part, can be corrected by interventions targeting mitophagy, autophagy, SOCS1 signaling, and the JAK-STAT pathway using a drug repurposing therapy approach [10,11,12,13]. The *CLEC16A*^ΔUBC^ mouse represents a unique model to study the role of *CLEC16A* and associated autophagy/mitophagy defects leading to autoinflammatory, lipodystrophy, and neurodegeneration (Figure 7).

Figure 7 depicts various drug targets to address the consequences of CLEC16A hypofunction, aimed at restoring cell homeostasis in response to mitochondrial dysfunction. (A) Natural compounds such as spermidine [138,139,140] and Urolithin-A an ellagitannins-derived metabolite have been shown to induce muscular mitophagy in cultured cells and *C. elegans* [141,142] and neuronal mitophagy in both *C. elegans* and the mouse brain [143,144]. (B) mTOR signaling with Rapamycin to stimulate mitophagy through AMPK and ULK1 activation [145,146,147,148,149,150]. (C) Supplementing with AICAR [151,152] and Metformin [153,154,155,156], an oral FDA-approved antidiabetic drug, can activate the enzymatic activity of AMPK, leading to ULK1 phosphorylation and activation. This, in turn, promotes autophagy initiation and the removal of damaged mitochondria. Additionally, AMPK-mediated activation of PGC-1α supports the promotion of mitochondrial biogenesis. The AMPK signaling pathway is a crucial regulator of cellular energy metabolism and has been demonstrated to induce autophagy and enhance mitochondrial function. (D, E, and F) Resveratrol (a polyphenolic compound, mainly found in red grapes skin, with beneficial properties for organismal homeostasis) [157,158], several synthetic drugs such as SIRT1720 and SIRT2104, and NAD+ suppliers indirectly enhance SIRT1 activity through AMPK activation, which raises cytoplasmic NAD^+^ levels and autophagy [159,160,161,162]. (G) Inflammation and cytokine signaling through SOCS-mediated JAK-STAT inhibitors. (H) Loss of *CLEC16A* leads to a vicious cycle of autophagic/mitophagy impairment and ER stress. Pharmacological inhibition of ER stress by any one of the three branches of unfolded protein response (UPR) namely Inositol Requiring 1 (IRE1), PKR-like ER kinase (PERK), and Activating Transcription Factor 6 (ATF6) could be beneficial. (I) Necrostatin-1 (Nec-1) is a potent and specific small-molecule inhibitor of receptor-interacting serine/threonine-protein kinase 1 (RIPK1). RIPK1, through its kinase and scaffolding functions, is a key regulator of apoptosis, necroptosis, and inflammatory pathways [163,164]. (J) The compound z-VAD-FMK (zVAD) is a cell-permeant pan-caspase inhibitor that irreversibly binds to the catalytic site of caspase proteases and can inhibit the induction of apoptosis [165].

These studies further support that therapeutic targeting of mitophagy holds promise for the treatment of autoimmune diseases as well as for Parkinson’s disease.

## 7. Conclusions

*CLEC16A* is emerging as a master regulator of autoimmunity and neurodegeneration. Overall, while the exact role of CLEC16A in health and disease is still being elucidated, it appears to play a critical role in the regulation of autophagy, mitophagy, and other cellular processes related to immune function, insulin secretion, and cellular homeostasis. Further fine mapping and biological studies of the 16p13 region are needed to fully understand the mechanisms involved and to identify effective therapeutic interventions in clinical settings. Drugs with modulatory effects on ER stress, lipophagy/autophagy/mitophagy, or inflammatory pathways could compensate for the attenuated CLEC16A activity and present formidable candidates for targeted interventions in autoimmunity and neurodegeneration. Interventions directed at reversing the dysregulated mitophagy and the consequence of loss of function of *CLEC16A*, including the use of repurposed drugs aimed at compensating for the attenuated CLEC16A activity, present formidable candidates for targeted interventions for autoimmune and neurodegenerative disorders.

## Figures and Tables

**Table 1:** Lead CLEC16A SNPs associated with 18 different autoimmune diseases.

Disease	SNP	Localization	Cases/Controls/Trios	Subject Cohort	Reference	PubMed Link (Accessed on 27 April 2023)
**Primary** **Adrenal** **Insufficiency (PAI)**	rs12917716	Intron 19	542/1220	Norway, UK	[60]	https://www.ncbi.nlm.nih.gov/pubmed/18593762
	rs12917716	Intron 19	479/2394	Sweden	[59]	https://pubmed.ncbi.nlm.nih.gov/29849176
**Allergic** **Rhinitis (AR)**	rs7203459	Intron 22	762/760	Han Chinese	[81]	https://pubmed.ncbi.nlm.nih.gov/32082391
	rs2286973	Exon 19	995/1004	Chinese population	[80]	https://pubmed.ncbi.nlm.nih.gov/36588789
	rs887864	Intron 19				
	rs12935657	Intron 21				
	rs11645657	Intron 22				
	rs36045143	Intron 22				
**Alopecia** **Areata (AA)**	rs998592	Intron 19	1702/1723	European ancestry	[67]	https://www.ncbi.nlm.nih.gov/pubmed/22534877
	rs3862469	Intron 19	2332/5233	European ancestry	[7]	www.ncbi.nlm.nih.gov/pubmed/25608926
**Asthma**	rs62026376	Intron 22	6685/14,091	European ancestry	[103]	www.ncbi.nlm.nih.gov/pubmed/24388013
	rs7203459	Intron 22	28,399/128,843	European ancestry	[79]	www.ncbi.nlm.nih.gov/pubmed/27182965
	rs9923856	Intron 19	1750/9245	African and European Americans	[78]	www.ncbi.nlm.nih.gov/pubmed/27611488
	rs62026377	Intron 22				
	rs17806299	Intron 19	19,954/107,715	European ancestry	[77]	www.ncbi.nlm.nih.gov/pubmed/29273806
	rs36045143	Intron 22	39,770/76,768	European ancestry	[76]	www.ncbi.nlm.nih.gov/pubmed/29785011
	rs12935657	Intron 21	40,544/300,671	European ancestry	[104]	www.ncbi.nlm.nih.gov/pubmed/30929738
	rs35441874	Intron 19				
	rs35032408	Intron 20	21,564/318,237	British ancestry	[75]	www.ncbi.nlm.nih.gov/pubmed/31036433
	rs7203459	Intron 22	5135/25,675	European ancestry	[74]	www.ncbi.nlm.nih.gov/pubmed/30552067
	rs35441874	Intron 19	46,802/347,481	European ancestry	[76]	www.ncbi.nlm.nih.gov/pubmed/31619474
	rs35441874	Intron 19	56,167/352,255	European ancestry	[73]	https://pubmed.ncbi.nlm.nih.gov/34103634
**Autoimmune** **Thyroid** **Diseases (ATD)**	rs2903692	Intron 22	330621	Japanese	[42]	https://pubmed.ncbi.nlm.nih.gov/18940880
	rs6498169	Intron 22	667/301	Chinese Han	[82]	https://www.ncbi.nlm.nih.gov/pubmed/24646814
**Celiac Disease**	rs12928822	intergenic	4,533/10,700	European populations	[85]	https://pubmed.ncbi.nlm.nih.gov/20190752
**Common** **Variable** **Immunodeficiency (CVID)**	rs17806056	Intron 19	778/10,999	Sweden, Norway, USA, UK, Germany	[83]	https://www.ncbi.nlm.nih.gov/pubmed/25891430
**Crohn’s** **Disease (CD)**	rs2903692	Intron 22	1264/890	Spain	[65]	https://www.ncbi.nlm.nih.gov/pubmed/19337309
**Eosinophilic Esophagitis (EE)**	rs12924112	Intron 21	1214/3734	European ancestry	[84]	https://pubmed.ncbi.nlm.nih.gov/29904099
**Juvenile** **Idiopathic** **Arthritis (JIA)**	rs6498169	Intron 22	1318/2149	Norway	[68]	https://www.ncbi.nlm.nih.gov/pubmed/19734133
**Multiple** **Sclerosis (MS)**	rs6498169	Intron 22	2322/5418/1540	European ancestry	[46]	www.ncbi.nlm.nih.gov/pubmed/17660530
	rs6498169	Intron 22	1146/1309	Australia	[47]	https://www.ncbi.nlm.nih.gov/pubmed/18650830
	rs11865121	Intron 19	2624/7220	European ancestry	[49]	www.ncbi.nlm.nih.gov/pubmed/19525953
	rs725613	Intron 19	1498/1706	Sardinia	[35]	https://www.ncbi.nlm.nih.gov/pubmed/18946483
	rs12708716	Intron 19	5737/10,296/ 2369	Australia, Belgium, Norway, Sweden, UK, USA	[50]	https://www.ncbi.nlm.nih.gov/pubmed/18987646
	rs6498169	Intron 19	1146/1309	Europe	[51]	https://www.ncbi.nlm.nih.gov/pubmed/19375175
	rs6498146	Intron 10				
	rs741177	Intron 18				
	rs876476	Intron 18				
	rs11863016	Intron 22				
	rs9937607	Intron 22				
	rs6498169	Intron 22	211/182 (+521 multiplex controls)	UK	[48]	https://www.ncbi.nlm.nih.gov/pubmed/19506219
	rs6498169	Intron 22	1853/2128	Holland, Canada	[105]	https://www.ncbi.nlm.nih.gov/pubmed/19834503
	rs6498169	Intron 22	435/550	Spain	[36]	https://www.ncbi.nlm.nih.gov/pubmed/19221398
	rs12708716	Intron 19	918/656	African Americans	[52]	https://www.ncbi.nlm.nih.gov/pubmed/19865102
	rs6498169	Intron 22				
	rs2080272	Intron 19				
	rs7200786	Intron 19	9772/16,849	European ancestry	[55]	www.ncbi.nlm.nih.gov/pubmed/21833088
	rs2041670	Intron 19	603/825	Europe	[53]	https://www.ncbi.nlm.nih.gov/pubmed/20849399
	rs998592	Intron 19	197/197	India	[54]	https://www.ncbi.nlm.nih.gov/pubmed/20952449
	rs12708716	Intron 19				
	rs12708716	Intron 19	3102/5047/1113	Norway, UK	[18]	https://www.ncbi.nlm.nih.gov/pubmed/21179112
	rs7206912	Intron 22				
	rs6498169	Intron 22				
	rs7184083	Intron 22	1343/1379	UK, US	[19]	https://www.ncbi.nlm.nih.gov/pubmed/21653641
	rs12927355	Intron 19	14,498/24,091	European ancestry	[56]	www.ncbi.nlm.nih.gov/pubmed/24076602
	rs4780346	Intragenic region				
	rs6498168	Intron 22	4888/10,395	German ancestry	[57]	www.ncbi.nlm.nih.gov/pubmed/27386562
	rs6498160	Intron 19	2273/2148	Sardinian	[58]	www.ncbi.nlm.nih.gov/pubmed/28445677
**Parkinson’s** **Disease (PD)**	rs7200786	Intron 19	342/503	European population	[87]	https://pubmed.ncbi.nlm.nih.gov/34149802
	rs6498169	Intron 22	515/504	Han Chinese	[88]	https://pubmed.ncbi.nlm.nih.gov/35432448
	rs12708716	Intron 19				
	rs12917716	Intron 19				
	rs7200786	Intron 19				
	rs2903692	Intron 19				
**Primary Biliary Cirrhosis (PBC)**	rs12924729	Intron 19	2460/7677	UK	[72]	www.ncbi.nlm.nih.gov/pubmed/21399635
	rs12708715	Intron 19	2861/8514	British and Irish ancestry	[70]	www.ncbi.nlm.nih.gov/pubmed/22961000
	rs12708715	Intron 19				http://www.ncbi.nlm.nih.gov/pubmed/22961000
	rs58102322	Intron 18	1450/2967	Europe	[71]	https://www.ncbi.nlm.nih.gov/pubmed/22257840
	rs12924129	Intron 19				
	rs12924729	Intron 19	2764/10,475	European ancestry	[69]	www.ncbi.nlm.nih.gov/pubmed/26394269
**Rheumatoid** **Arthritis (RA)**	rs6498169	Intron 22	600/550	Spain	[36]	https://www.ncbi.nlm.nih.gov/pubmed/19221398
	rs6498169	Intron 22	1318/2149	Norway	[68]	https://www.ncbi.nlm.nih.gov/pubmed/19734133
**Selective IgA** **Deficiency**	rs34069391	Intron 19	1635/4852	European ancestry	[66]	www.ncbi.nlm.nih.gov/pubmed/27723758
**Systemic** **Lupus** **Erythematosus (SLE)**	rs12599402	Intron 19	1656/3394	Han Chinese	[64]	www.ncbi.nlm.nih.gov/pubmed/23273568
	rs7200786	Intron 19	5201/9066	European ancestry	[63]	www.ncbi.nlm.nih.gov/pubmed/26502338
	rs9652601	Intron 19				
	rs9652601	Intron 19	5695/10,352	Chinese and European ancestry	[62]	www.ncbi.nlm.nih.gov/pubmed/27399966
	rs9652601	Intron 19	6748/11,516	European ancestry	[61]	www.ncbi.nlm.nih.gov/pubmed/28714469
	rs2041670	Intron 19				
	rs8054198	UTR				
**Systemic** **Sclerosis**	rs1646066	intergenic	291/260	African Americans	[86]	https://pubmed.ncbi.nlm.nih.gov/29293537
**Type 1** **Diabetes** **(T1D)**	rs12708716	Intron 19	2000/3000	European ancestry	[34]	www.ncbi.nlm.nih.gov/pubmed/17554260
	rs12708716	Intron 19	1963/2938	European ancestry	[32]	www.ncbi.nlm.nih.gov/pubmed/17554300
	rs725613	Intron 19	1896/1146/873	European ancestry	[1]	www.ncbi.nlm.nih.gov/pubmed/17632545
	rs2903692	Intron 22				
	rs17673553	Intron 22				
	rs12708716	Intron 19	3561/4646	European ancestry	[33]	www.ncbi.nlm.nih.gov/pubmed/18978792
	rs12708716	Intron 19	7514/9045	European ancestry	[43]	www.ncbi.nlm.nih.gov/pubmed/19430480
	rs2903692	Intron 22	735/621	Japan	[42]	https://www.ncbi.nlm.nih.gov/pubmed/18940880
	rs725613	Intron 19	1037/1706	Italy (Sardinia)	[35]	https://www.ncbi.nlm.nih.gov/pubmed/18946483
	rs725613	Intron 19	205/422	China (Han)	[39]	https://www.ncbi.nlm.nih.gov/pubmed/19178520
	rs6498169	Intron 22	316/550	Spain	[36]	https://pubmed.ncbi.nlm.nih.gov/19221398
	rs12708716	Intron 19	1212/2513	Germany	[41]	https://www.ncbi.nlm.nih.gov/pubmed/21873553
	rs2903692	Intron 22	1743/790	Japan	[40]	https://www.ncbi.nlm.nih.gov/pubmed/22069271
	rs12708716	Intron 19	8506/10,596	European ancestry	[44]	www.ncbi.nlm.nih.gov/pubmed/21829393
	rs12921922	Intron 4	131/121	China	[38]	https://www.ncbi.nlm.nih.gov/pubmed/22778732
	rs12931878	Intron 1				
	rs12927355	Intron 19	6683/12,173/69 and 2601 affected sibling pair families	European ancestry	[45]	www.ncbi.nlm.nih.gov/pubmed/25751624

## Data Availability

All data, supporting opinions, and conclusions reported in the current manuscript are available at https://pubmed.ncbi.nlm.nih.gov and https://genome.ucsc.edu.
